# Combating Lassa Fever in West African Sub-Region: Progress, Challenges, and Future Perspectives

**DOI:** 10.3390/v15010146

**Published:** 2023-01-03

**Authors:** Chinyere Aloke, Nwogo Ajuka Obasi, Patrick Maduabuchi Aja, Chinedum Uche Emelike, Chinedu Ogbonnia Egwu, Olamide Jeje, Chuks Oswald Edeogu, Olalekan Olugbenga Onisuru, Obasi Uche Orji, Ikechukwu Achilonu

**Affiliations:** 1 Protein Structure-Function and Research Unit, School of Molecular and Cell Biology, Faculty of Science, University of the Witwatersrand, Braamfontein, Johannesburg 2050, South Africa; 2 Department of Medical Biochemistry, Alex Ekwueme Federal University Ndufu-Alike, Abakaliki PMB 1010, Ebonyi State, Nigeria; 3 Department of Biochemistry, Faculty of Biological Sciences, Ebonyi State University, Abakaliki PMB 053, Ebonyi State, Nigeria; 4 Department of Biochemistry, Faculty of Medicine, Mbarara University of Science and Technology (MUST), Mbarara P.O. Box 1410, Uganda; 5 Department of Medical Biochemistry, Kampala International University, Bushenyi, Ishaka P.O. Box 71, Uganda; 6 Department of Physiology, Alex Ekwueme Federal University Ndufu-Alike, Abakaliki PMB 1010, Ebonyi State, Nigeria; 7 Department of Medical Biochemistry, Faculty of Basic Medical Sciences, Ebonyi State University, Abakaliki PMB 053, Ebonyi State, Nigeria

**Keywords:** Lassa fever, nucleoprotein, ribavirin, Lassa virus, target validation

## Abstract

Lassa fever (LF) is a rodent-borne disease that threatens human health in the sub-region of West Africa where the zoonotic host of Lassa virus (LASV) is predominant. Currently, treatment options for LF are limited and since no preventive vaccine is approved for its infectivity, there is a high mortality rate in endemic areas. This narrative review explores the transmission, pathogenicity of LASV, advances, and challenges of different treatment options. Our findings indicate that genetic diversity among the different strains of LASV and their ability to circumvent the immune system poses a critical challenge to the development of LASV vaccines/therapeutics. Thus, understanding the biochemistry, physiology and genetic polymorphism of LASV, mechanism of evading host immunity are essential for development of effective LASV vaccines/therapeutics to combat this lethal viral disease. The LASV nucleoprotein (NP) is a novel target for therapeutics as it functions significantly in several aspects of the viral life cycle. Consequently, LASV NP inhibitors could be employed as effective therapeutics as they will potentially inhibit LASV replication. Effective preventive control measures, vaccine development, target validation, and repurposing of existing drugs, such as ribavirin, using activity or in silico-based and computational bioinformatics, would aid in the development of novel drugs for LF management.

## 1. Introduction

Lassa fever (LF) is an acute viral hemorrhagic fever ailment that can degenerate into neurological diseases [1]. Reports have shown that the Lassa Virus (LASV) which is the causative agent for LF is predominant in several countries in West Africa [2]. The disease is endemic in this region and has been reported to have affected over 2 million people. Studies have shown that about 300,000 to 500,000 cases are diagnosed, and 5000 mortalities are recorded annually [3]. Reports indicated an increase in the prevalence of LF as a result of the movement of people from LF endemic zones to non-endemic areas, including its spread through air [4] and a high rate of seroprevalence in Sierra Leone, Nigeria, and Guinea including non-endemic regions [5]. LASV is an RNA virus enveloped in a lipid membrane. The RNA genome of the virus is bi-segmented and its coding strategy is in the ambisense form. On the surface of the virus is the envelope glycoprotein complex (GP) which appears as trimeric spikes and serves as a prime target for the design of antibody-based vaccines and therapeutics [6]. LASV is an arenavirus. Arenaviruses are grouped into two: based on genetic differences and geographic distribution. The first group, New World viruses are found in the Western Hemisphere—North and South America while the second group, Old World viruses are found in the Eastern Hemisphere—Africa, Europe, and Asia. Lymphocytic choriomeningitis virus (LCMV) which belongs to Old World arenavirus, is the only arenavirus found in both the Western and Eastern Hemisphere [1,2]

LF is predominant in most West African sub-regions with its incidence more prominent in Guinea, Liberia, Nigeria, and Sierra Leone because *Mastomys natalensis,* the animal which serves as LASV reservoir and vector is more abundantly seen in these regions [7]. It has orchestrated significant hardship in the economy of these countries and poses a worldwide clinical challenge for the public officials owing to the danger of importation of LF patients [8,9]. LASV has an incubation period between 6 to 21 days, which is relatively long and thus, places it among the most common exported viral haemorrhagic fevers (VHFs) to non-endemic countries. This attribute prompts international interest in LASV on global health security [5]. As a result of its high case fatality rate, potential to spread easily via human-human contact, severity of infectivity, lack of effective vaccines and therapeutics and spread via aerosol, it is classified as Biosafety Level 4 agent [10,11]. Contrastingly, the contributions of LASV to the increased threat of viral diseases in West Africa have been poorly estimated, although it accounts for significant causes of yearly morbidity and mortality in most of Africa’s poverty-stricken communities [12,13].

Currently, there is an increase in awareness that LF is a significant health challenge. This became clearafter the 2013–2016 Ebola virus disease (EVD) epidemic in the Mano River Union (MRU) countries including Sierra Leone, Liberia, and Guinea. This epidemic spurred international agencies to respond and facilitate the prediction of viral disease outbreaks in West Africa. In 2015, the World Health Organization (WHO) reported that LF is among the priority ailments needing urgent attention for research and development [14,15]. Consequently, LASV was listed as a priority for research funding by the multi-agency Coalition for Epidemic Preparedness Innovations (CEPI), in addition to many other emerging viruses for the development of vaccine [16].

At present, no approval has been granted for a therapeutic approach or prophylactic vaccine for LASV, making it a severe health challenge for humans in endemic countries. Ribavirin, a nucleoside analog has been reported to be efficacious at the onset of infection the drug is linked with severe side effects [17]. Recent outbreaks in Nigeria with high fatality rates accentuate the urgent need for the acceleration of research activities aimed at the development of licensed therapeutics and vaccines [18]. The rising numbers of LF cases reported in the endemic sub-regions of West Africa have caused serious alarm over the exportation of the virus to non-endemic countries in the region [19]. More than 35 cases of LF have been reported as exported cases from the first case in 1969 until 2020. Reports from the literature have shown that reported cases of LF were transported internationally from seven countries in the West African region to nine countries situated in Europe, Asia, South Africa, and North America [9,20]. Recently, the United Kingdom health authorities notified WHO of two laboratory confirmed cases and one probable case of LF on the 9th of February 2022. Three confirmed cases and one death were reported as of 18th February 2022 [21]. These were believed to be due to human-to-human transmission as the first case travelled to Mali where LF is endemic in late 2021, thus accentuating the risk of export from endemic countries. Many people have been deported, and air movement restricted owing to the danger of spreading LF during its breakout in endemic regions or exposure during medical procedures [22,23].

Even though LF is endemic in African countries such as Nigeria, the details of outbreak and containment have not been properly documented in these regions. Thus, it is germane to learn to keep appropriate records from past experiences to improve in the handling and containment of future outbreaks [24]. This is because of the paucity of information concerning the control and handling of cases and/or suspect tracking during outbreaks scenarios. There has been rising concern on the potential of LASV as a biological weapon, still, no lasting remedy to this clinical challenge has been developed so many years after its outbreak in remote villages in countries such as Nigeria [25]. Thus, this review explores the pathogenesis and epidemiology of LF, its management and appraises the associated health problems and the hurdles in controlling the epidemic and future perspectives on prime focus.

## 2. Epidemiology of Lassa Fever in West African Countries

### 2.1. Prevalence

LF which is orchestrated by the LASV of the Arenaviruses family, is a resurgence challenge in the public health sector that has led to an increase from about 100,000 to 300,000 reported cases in West Africa regions per annum with 5000 mortalities annually [26]. Reports have shown that about one percent of LF cases culminate in mortality [26,27]. Reported LF cases in Sierra Leone and Liberia showed that 10–16% of patients in hospitals annually have LF. This shows that these countries are highly endemic [28]. Africa Centre for Disease Control (CDC) (2018) in her report confirmed a total of 1,182 cases of LF in Nigeria, Liberia, and the Benin Republic between 2016–2018. In Nigeria, reports showed that a total of 1615 LF cases were confirmed in 2021 [28]. Additionally, in 2019, 578 confirmed cases were reported in Nigeria [29].

### 2.2. Epidemiology

In the 1950s, LF disease was described first in Sierra Leone even though the causative agent (virus) was not established. In 1969, two nurses who were on missionary assignment reportedly died in Nigeria, and the ailment that led to their death was discovered to be LF caused by LASV, named after the Nigerian town (Lassa in the Yedseram River valley) wherein the first cases were identified [30]. LF is an animal-borne acute ailment caused by LASV. LASV is an arenavirus responsible for a serious hemorrhagic fever symptomatic of high fever, vomiting, sore throat, muscle aches, nausea, chest, and abdominal pain [31]. Reports have shown that the period of incubation for LF ranges from 6–21 days. The clinical manifestations of LF are unspecific, and this makes it very difficult at the early stage of the disease to differentiate it from other common febrile diseases like malaria [27]. At the severe stage, LF may show as a viral hemorrhagic fever causing shock and multi-organ malfunction that may lead to death. Generally, about 80 percent of LF cases are asymptomatic or may present subclinical symptoms that are not easily recognized clinically [26,27].

Currently, reports show that there is annual breakout of LF in countries in the West African zone with the greatest incidence of occurrence in Nigeria. Lassa hemorrhagic fever occurs with seasonal spikes mostly during the dry season (November to April), and it is predominant in Nigeria, Sierra Leone, Mali, Ghana, Benin, Togo, Guinea, and Liberia [32]. Furthermore, LF is a location-specific outbreak with high mortality rate [26]. As a result, early detection of infected patients is critical for the prompt implementation of carrier isolation nursing guidelines [26]. The virus infection is asymptomatic but persistent with high urinary viral excretion in *Mastomys natalensis*, the most common commensal rodent host [29]. Increased inter-border traffic and international travel have given rise to more virulent and contagious LASV in many communities in the endemic countries within the sub-region of West Africa and also increased the sporadic cases of the fever in non-endemic regions of Africa and other continents [33]. Thus, it is necessary for relevant health officers to have all information relating to the virus and its infectivity.

### 2.3. Transmission of LF

LF is a disease of animal origin, and the LASV shows persistent infection accompanied with profuse excretion of the virus in urine by the rodent, *Mastomys natalensis* that is ubiquitous [33]. The breakout of LF in endemic regions is usually spurred by any activity or factors that enhance the contact between human and rodent. These factors are sanitation, overcrowding, cutting down of trees, hunting of rodents, social upheaval, bush burning, as well as agricultural activities like the cultivation of staples foods for zoonotic animals [12]. In the West African sub-region, rural settlers are vulnerable to LF as a result of their closeness to the zoonotic host, mode of settlement which involves the building of open villages, drying of grains by the roadside, or its storage at locations accessible to rodents. Consequently, these conditions promote increased rodent-human contact or food contamination by the secretions of the rodents such as urine or faces [34]. It is also transmitted via contamination of bodily fluid of infected person or a reservoir host carrying the virus [7]. The virus is capable of circulating within the community without detection [7].

Furthermore, it has been reported that suppression of the immune system due to a particular underlying transmissible or non-transmissible illnesses, chemotherapy, in addition to pregnancy (especially at third trimester) could promote infection and establishment of LF and increase the death rate, raising it to 80% [35]. Fetal death can arise during pregnancy if the pregnant woman is infected with LF (because the virus has a strong attraction for highly vascularized tissues such as the placenta) through premature abortion or death of the newborn (about 90 percent of cases) or maternal death [13,36]. Children born with LF infections experience severe congenital defects or anomalies [37].

A recent study indicated that the LASV is also harbored by other rodent species like *Hylomyscus pamfi* (African wood mouse) and *Mastomys erythroleucus* (Guinea multimammate mouse) in Nigeria and Guinea, respectively [38]. The transmission of LF from rodents to humans occurs through having contact with carrier rodents or its contaminated excreta such as urine, feces, or nasal discharges [13]. Furthermore, the infection of LASV can equally be gotten via ruptured skin or through mucous membrane exposure to contaminated material or item [33,35]. Furthermore, LASV infection can also be obtained via nosocomial acquisition, particularly by contact with an infected, unprotected hospital personnel, or contaminated blood [35]. In the same vein, other suggested possible means of infection include direct contact with semen infected with LASV, virginal exudates in addition to intake of infected breast milk [37].

## 3. Clinical Perspectives

### 3.1. Pathology

There is a limitation in the current knowledge about the pathology of LF as a result of social upheaval, societal stigma, and practices associated with the dead in the endemic regions [39]. An earlier report on the pathologic investigation of reported cases in human subjects after post-mortem examination shows injury in the hepatocyte, adrenal glands as well as in the spleen [40]. Other studies on the analyzed histologic results of the hepatocyte revealed marked necrotic eosinophils and parenchymal cells accompanied by penetration of eosinophils in the sinusoids, while analysis of spleen samples shows necrosis of eosinophils, depletion of lymphoid, deposition of fibrin, and shrinking of the white pulp as well as infiltration of mononuclear cells and lymphocytes [40,41,42]. Previous individual study on the analysis of adrenal gland samples in human indicated multiple foci necrosis of adrenocortical cells often linked with swelling [40]. Other minor findings were petechiae of the gastrointestinal tract, renal edema, damage to renal tubule, interstitial nephritis, lymph node sinus histiocytosis, light interstitial pneumonia, hemorrhage, and mild interstitial mononuclear myocarditis [40,41,42]. However, no changes were observed in the mammary, placenta, uterine, pancreas, ovarian, central nervous system, and brain samples of the human subject [40].

### 3.2. Pathogenesis of LF

LASV is a member of the Arenaviruses (AV) family having a single-stranded negative ribonucleic acid (RNA) [7]. The virus has two-segmented ribonucleic acid, a nucleoprotein, a lipid envelope, and a glycoprotein as its most salient parts [5]. The virus employs alpha-dystroglycan in establishing itself into targeted cells mostly macrophages, dendritic, and endothelial cells which is the point of commencement of its replication [5]. LASV prevents interferon manufacturing by the infected cell via its nucleoprotein. In addition, LASV subdues the cells of the immune system thus preventing their secretion of proinflammatory cytokines including tumor necrosis factor (TNF)-α, IL-6, and IL-8β, unlike the symptoms observed in other hemorrhagic fevers [5].

There is an initiation of LF infection in an individual on the acquisition of LASV via body touch with carrier rat excreta (urine, saliva, and respiratory secretion) or blood. The antigen-presenting cells are the focal points of the virus immediately after it gets into the host cells. The virus infects most tissues in humans culminating in multi-systemic malfunction, immunosuppression of host’s innate interferon (IFN) response via halting of interferon regulatory factor-3 (IRF-3) translocation [43,44]. Reports have shown that LASV has exonuclease activity only targeted at double-stranded RNAs, which mostly inhibits the responses of IFN. This is achievable via assimilation of pathogen-associated molecular patterns (PAMP), which helps the LASV to circumvent the immune response of the host [45]. The blood vessels are the tissues mostly afflicted and the LASV replicates in the cells of the reticuloendothelial system culminating in capillary injury. Bleeding might be observed in the following organs such as hepatocyte, intestine, myocardium, lungs as well as the brain [33,46].

Additionally, infection-triggered induction of unregulated expression of cytokine akin to what is seen in sepsis could be another possible mechanism of LF pathogenesis. There is documented evidence of the occurrence of fatal LF that was imported into Germany in the year 2000 [35]. In the report, the sufferer passed on due to failure of multiple organs and shock due to hemorrhage with clinical findings showing elevated concentrations of proinflammatory cytokines, interferon γ (IFN- γ), and tumor necrosis factor α (TNF- α) before death. However, no elevation in both cytokines was observed in another study of LF lethal cases, indicating that IFN- γ and TNF- α concentrations are either increased only in a fragment of infected individuals or within a brief duration that could entail incessant testing for detection [47].

Furthermore, the suppression of immune response orchestrated by the virus might be another possible mechanism implicated in the pathogenesis of LF [48]. Activation of dendritic cells (DC) and macrophages (MP) obtained from monocyte and human, respectively, fails on infection with LASV. There is a malfunction in the infected DC culminating in failure in the secretion of proinflammatory cytokines, upregulation of costimulatory molecules including CD40, CD80, and CD86, and abysmal induction of T cells’ growth [49,50]. In another study, human DC infected with Mopeia virus (nonpathogenic) which is a similar arenavirus that has 75 percent resemblance in amino acid sequence with LASV and was obtained from the same rat reservoir [51], causes stronger induction in the responses of CD4 and CD8 T-cell unlike patients with LASV [52]. Repression of the immunologic reactions orchestrated by LASV contamination revealed ex vivo is equally in tandem with the outcomes of medical examination, indicating that lethal result of LF is associated with reduced concentrations or paucity of interleukin (IL) 8 and IFN inducible protein 10 (IP-10) in circulation [47].

### 3.3. Morbidity and Mortality of LF

LF is an ailment of public health importance owing to its morbidity and associated mortality as well as its capability for residual morbidities like loss of hearing and social stigma. An understanding of the number of deaths among LF patients is one way of evaluating the efficacy of the recent strategies used in its management [53].

Abundant evidence has shown that about 15–20% of LF-infected hospitalized subjects die from the diseases; although, approximately 80% of humans infected with LASV are asymptomatic or presents sub-clinical symptoms, and one percent culminate in death [28,54,55]. Additionally, LASV has been implicated to cause high fetal death and high death rate among pregnant women [36]. In early pregnancy, the death rate for fetuses is 92%, about 75 percent for unborn babies in the third trimester, and 100 percent for children in the first four weeks of their life while the death rate for expectant mothers is 7 percent within the first two months, 30 percent in the last trimester and 50 percent of expectant mothers who will birth within one month. Contrastingly, the mortality rate generally for women without pregnancy is 13 percent [36,56]. In a study by Kernéis et al. [7], seroprevalence of 12.9 and 10.0 percent in the village and urban participants, respectively, indicating prior exposure to LASV was reported. Out of those with positive serology, 13 percent of them did not display any clinical sign in the previous 12 months which is evidence corroborating the fact that LASV infection can be asymptomatic.

Robust evidence indicates that delay in looking for medical care had a negative impact on the survival of LF patients [57,58,59]. Earlier reports indicated that ribavirin is efficacious if the drug is given at the early stage of infection [58,60]. Age is an important predictor of patients’ survival in LF. The mortality rate of LF is linked with increasing age [61]. However, higher mortality among younger and older patients has been reported [62]. Underlying medical conditions like pregnancy is an important index of treatment outcome in LF. LF-infected pregnant females respond to treatment poorly [36,60]. In the same vein, the fetal outcome is severely affected in LF [36,60]. Previous study has shown a high incidence of acute kidney injury (28 percent) in LF and this is an indication of poor treatment outcome [61]. Proper coordination and response during LF outbreak and establishment of infection prevention control measures might aid in curtailing LF-associated morbidity and mortality [62].

**Figure 1:**
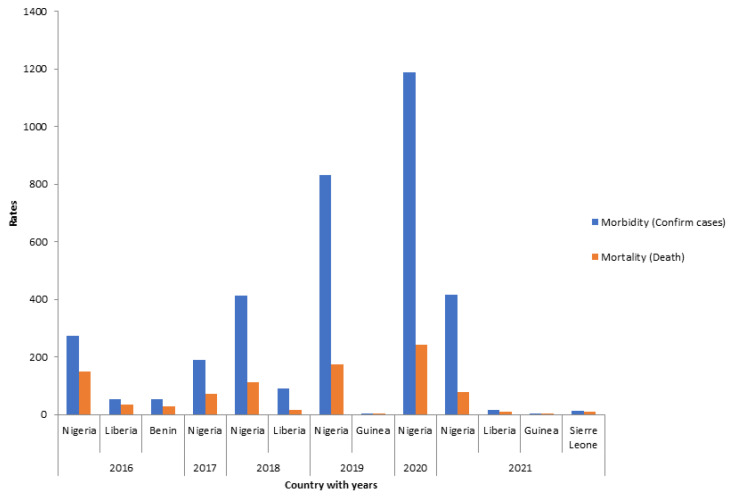
Morbidity and mortality rate in West Africa from 2016–2021 [28,63].

Reports have shown that the case presentation is seasonal with peak periods at the dry season and nadir periods at the wet season [12]. The morbidity and mortality rates from 2016 to 2021 in the most affected countries are shown in Figure 1.

### 3.4. Clinical Manifestations

The mode of clinical manifestation of LF could be unspecific and this poses a problem for some cases during clinical investigation. The zoonotic host though carrying the virus does not show symptoms but sheds viruses in the waste matter such as urine and feces. About 80% of subjects infected with the LF do not display any symptoms of the disease [12,64]. Nevertheless, clinical signs in individuals infected differ and range from acute and severe fatal hemorrhagic fever, accompanied by multiple organ failures as seen in the liver, spleen, and kidney [13,65]. The symptoms of LF fever resemble what is observed in other ailments such as malaria and typhoid prevalent in the area and hence can be confusing. This ambiguity in the nature of the symptoms poses a clinical challenge in the detection of infected individuals [61,66].

Multiorgan failure often led to death within fourteen days of visible symptoms [67]. The long-established aftereffect like sensorineural hearing losses is provoking a massive social-economic burden across the West African region, where stigmatization and isolation culminate in the increment in the rates of unemployment and depression [68]. In Nigeria alone, aid programs can cost up to $43 million annually [69].

Clinical investigations of the throat of infected patients are usually indicative of exudative pharyngitis while analysis of urine samples is usually marked by the presence of protein in the urine. There is always diminution in neutrophil count. At this early stage, neurological signs such as tremors, convulsions, and meningitis are not usually visible. However, sensorineural hearing loss sometimes manifests. Previous studies indicate that observance of timely sensorineural hearing damage and likely presentations of other central nervous system characteristics demonstrate implausible prognostication [70,71]. Robust evidence from 441 patients revealed that the best index of LF is the aggregation of fever, pharyngitis, retrosternal pain, and proteinuria with a predictive value of 0.81. The best indicator of the outcome is an aggregation of fever, sore throat, and vomiting with a risk ratio of death value of 5.5 [72]. Additionally, in this study, bleeding in the mucosa, deafness in the bilateral or unilateral eighth-nerve deafness, or effusion of the pericardia were reported as 17%, 4%, and 2%, respectively. In a similar study carried out in Nigeria with 908 patients, other factors such as societal habits and sanitation were seen as formidable elements enhancing the dissemination of the disease [73].

Infected individuals with no noticeable clinical symptoms of LF go about unrecognized. Although this category of people together with those that survived the acute LF ailment are prone to the development of loss in hearing of varying degrees later in life. The loss in hearing is normally bilateral and could influence all degrees of hearing [70] and approximately 25 percent of individuals exposed to LAVS are affected [74]. The origin and development of this loss in hearing is speculated to arise from an immunological reaction between the circling LASV immunoglobins and the plasma membrane at the basal side of the cell/cochlear outer hair cells [73]. Yun et al. [75] have recently established a murine model for LF that closely mimics several characteristics of LF ailment in human. Using this model, the LASV obtained from a lethal human case was highly virulent and the virus obtained from a non-lethal case elicited mostly mild disease with moderate mortality but both viruses induced SNHL in surviving animals. Additionally, in a recent study conducted by Maruyama et al. [76] in mouse LF model in which they evaluated the auditory function employing auditory brainstem response (ABR) and distortion product otoacoustic emissions (DPOAE) in determination of the underlying mechanisms of LASV-induced hearing loss; they pioneered measures of ABR and DPOAE tests in rodents in biosafety level 4 (BSL-4) facilities and concluded that depletion of T cell indicated that CD4 T-cells play crucial function in hearing loss induced by LASV.

## 4. Management of LF

Currently, there is no licensed prophylactic drug or therapy for LF, supportive care with symptoms management has been the recommended course for clinical management. The prime objective is volume rejuvenation, contributing to diarrhea, third spacing, and vomiting, as well as avoidance of volume overload, owing to the danger of accumulation of fluids in the lung. Additional objectives include homeostasis of electrolyte and respiratory support [77]. Ribavirin treatment is limited in the management of the ailment with different levels of clinical effectiveness [64,78]. However, studies have shown that timely administration of ribavirin at the inception of symptoms is efficacious in the treatment of LF. Thus, administration of ribavirin *per os* has been employed for after exposure prophylaxis for individuals prone to secondary infection [24,79,80,81]. Although, about four different LF virus lineages namely I, II, III, and IV have been reported with Nigeria having lineages I-III in common while lineage IV is peculiar to Sierra Leone [82]. This diversity in genetic variation is believed to be a major setback facing the development of vaccines and also aids in underestimating its occurrence [78,83,84,85]. Previous studies have shown observable diminution in the number of deaths for severe cases. In the same vein, towering death rates have been seen for patients treated with ribavirin and those not treated which emphasizes the importance of a better remedy for Lassa management [60,86].

Mounting evidence has shown that ribavirin’s precise mechanism of action against LASV is yet to be fully understood. However, studies suggested the integration of drugs into the RNA strand, culminating in the lethal genetic mutation of progeny genomes [87,88,89]. Its advantage was more pronounced in vulnerable subjects with the elevated virus in the bloodstream and liver status test, and when administered within six days from the inception of symptoms. Administration of ribavirin orally and intravenously was beneficial, though intravenous administration has a more pronounced effect in higher risk patients [5]. The usage of ribavirin in LF management is widely well accepted, however, reversible hemolytic anemia has been observed frequently as a negative outcome of the treatment that might warrant adjustment of dosage or treatment discontinuation in serious cases [90,91].

The ribavirin action mechanism is thought to occur via manifold means. Its transformation product, ribavirin monophosphate (RMP) has been suggested as among the principal effective configuration of the drug [92]. Studies have revealed that RMP hinders the action of inosine monophosphate dehydrogenase ((IMPDH), an enzyme required for guanosine triphosphate (GTP) synthesis, culminating in interference in the critical steps in viral replication [92,93,94,95]. Furthermore, another metabolite of ribavirin, ribavirin triphosphate (RTP) has been reported to function as a nucleoside analog and cause mutation via its integration into the genetic make-up of the virus by the viral RNA polymerase. It is believed that this accumulation of mutations inside the virus genome culminates in the repression of its replication via a phenomenon referred to as genomic catastrophe [87,96].

RMP and RTP actions had been hypothesized to be synergistic, with RMP causing a diminution in the accessibility of GTP via the halting of IMPDH and consequently reducing rivalry for incorporation of the mutagenic RTP by the viral RNA polymerase [87]. Alternatively, the antiviral activity of ribavirin may also be ascribed to its reduction in mortality in LASV infected cells via suppression of activation of macrophage, synthesis of cytokine, and lymphocyte proliferation [97].

Another drug that has demonstrated efficacy is a broad-spectrum RNA suppressor licensed for the management of influenza known as Favipiravir. This drug has wide-ranging activity as opposed to RNA viruses and has been revealed to reduce the blood level of LASV in animal models [98,99,100,101,102]. Report has shown that a high dosage of favipiravir (300 mg/kg daily for 2 weeks) was potent in the treatment of Lassa virus–viremic macaques unlike low multiple doses (50 mg/kg every 8 h) [102]. This animal model cynomolgus macaques; reliably recapitulates many signs of LF infection in humans [103]. High dose treatment with favipiravir in macaques was reported to successfully abate the pathophysiologic variables linked with LASV infection, culminating in survival. Thus, treatment outcomes indicated that little therapeutic action is obtained using small doses of favipiravir, while higher doses are more efficacious and will enhance clinical outcomes.

Favipiravir when administered goes through phosphoribosylation culminating in the transformation of the biologically inactive form of favipiravir to pharmacologically active favipiravir-RTP (favipiravir ribofuranosyl-50-triphosphate) that is accountable for its effect against virus [104,105,106]. The drug mechanistically imitates a purine nucleotide and it is integrated into the RNA of the virus in the course of replication or attaches to preserved areas of the enzyme, RdRp culminating in the termination of the transcription of the virus [104,105,107]. Genomic catastrophe is another promising mechanism akin to ribavirin, culminating in the increment in frequencies of mutation, particularly for G to A and C to T transitions [108]. Previous studies indicated that the wide-range effect exhibited by favipiravir in opposition to the viruses’ RNA may be elucidated by the comparatively preserved catalytic site of the RdRp amidst many of such viruses [104].

Investigation with animal models like guinea pigs and non-human primates (NHPs), using convalescent plasma (CP) having high titers of neutralizing antibodies has demonstrated promising results. Likewise, human monoclonal antibody (mab) cocktails having a strong binding attraction to glycoprotein complexes of Lassa virus lineages II, III and IV have been revealed to protect crossbred guinea pigs when given directly after challenge [86,109,110]. A previous study indicated that a cocktail of human mab protected non-human primates from LASV threat even when administered up to 8 days after infection with the Lassa virus. Remarkably, the cocktail immunoglobulins identify the epitomes of LASV- glycoprotein and bind glycoprotein derived from clades I-IV, culminating in the deduction that such immunoglobulins might possess the capacity for a cross-protective potential for Lassa virus in West Africa [78].

Convalescent sera have shown efficacy in the reduction in deaths among Argentine hemorrhagic fever patients in New World arenaviruses, like Junín virus [111,112]. Although, mixed results have been reported in human LF patients treated using CP (~3–4 mL/kg), and it has been proposed that matching of CP geographically, likewise the titers of nullifying antibodies per curative dosage, might be important elements in efficacious passive therapy [17,113,114,115]. The level of safety and in vivo repression of LASV in guinea pig animal models might be linked with nullifying potential (via PRNT and LNI) [109,113]. Furthermore, worries exist that the degree of counteracting effect in CP obtained from donors may be time-based (≥6 months after contamination needed for triggering of robust counteracting antibody titers) and that the concentration of unselective CP ought to be increased to be pharmaceutically effective [113]. Table 1 below summarizes previous studies/trials and details of possible treatments/therapies.

### 4.1. Prevention/Control

The transmission and acquisition of LF can be prevented or controlled by the implementation of some measures including policies formulation, the establishment of a task force and committees for monitoring, checkmating, and curtailing of LF nationally and at state levels. In addition, sensitization/health education of both health workers and the general public on the disease dynamics, transmissibility, symptoms, and preventive measures [34]. The ailment could equally be controlled by regulating the zoonotic host by the avoidance of bush burning, use of snares in and within homes to curtail their numbers, obstruction of rodents’ hiding place, and shunning rat hunting for human consumption.

Other preventive measures include good and healthy personal hygiene, good environmental sanitation, proper disposal of waste far-off from homes, avoidance of food spreading by the roadside or where rats will gain access to it as well as food-items storage in rat-proof containers [34]. In the hospital, the preventive strategies to be employed include adhering to contamination control guidelines, quarantine of contaminated individuals, barrier nursing of subjects contaminated, and usage of personal protective devices when attending to infected subjects or working with their fluids [34].

### 4.2. Vaccination as Feasible Control Measures for LF Curtailment

Although there is no approved vaccine currently for LF management, but vaccination represents the most feasible control measure. Few vaccine candidates have passed through the clinical stage of testing, as most of them are still in preclinical or early stages of clinical development. The vaccine forms which are in preclinical development include but not limited to measles virus-based vaccine (INO-4500), [that is in phase 2 clinical trials], MV-LASV (V182-001) [that is in phase 1 clinical trials], adenovirus vector-based (ChAd3, ChAdOx 1, and ChAd63), live-attenuated (ML29 and rLASV-GPC/CD), VSV-based, recombinant vaccinia-based (VAC-LASV), as well as RNA replicon LF candidate vaccines. The challenge with many of the vaccines in preclinical development is that many of them require the usage of more than one dose of vaccination. Several vaccination doses pose a challenge in underdeveloped countries as there is inadequate resources as well as lack of access to health care facility, and/or personal motivation [128]

Accumulating evidence suggest that there exist numerous LF candidate vaccines in preclinical stage of development, although no FDA-approved vaccines is currently in use for human LF treatment. There was emphasis by the 2017 WHO Target Product Profile (TPP) for LF vaccines stating that a high priority for prophylactic vaccines development, optimal candidates should meet acceptable standard for safety/reactogenicity as stipulated by WHO, including being single-dose as well as greater than or equal to 70% efficacy in preventing infection or disease orchestrated by the LASV lineages I-IV. Besides, it should be long lasting (≥5 years) [129,130]. Majority of the LF vaccines techniques and platforms are dependent on the LASV GP antigen from the Josiah strain and hence confer protection against a homologous virus challenge only [131,132]. In terms of effectiveness, cellular immunity is more efficacious in LASV infection clearance in comparison to humoral immunity, and so, GP and NP antigens have been selected for formulations of vaccine, particularly in viral vector-based and live-attenuated vaccine platforms [133,134].

One of the candidate vaccines in clinical trial is INO-4500. It is a DNA-based vaccine candidate which expresses the LASV GPC and is based on the Schwarz strain of the measles virus (MV) [135,136]. It entered the 1st phase of human clinical trial that investigated the safety, tolerability, and immunogenicity of the LF vaccine candidate in volunteers that are healthy and the study was completed in October of 2021, but the outcome is yet not available. The second stage of the clinical trial that investigated the dose-ranging in healthy volunteers, commenced in January 2021 and was expected to be completed in January 2022 [135].

Furthermore, MV-LASV (V182-001, MeV-NP) is another vaccine candidate which has been developed using the Schwarz MV vaccine platform besides INO-4500. It expresses the LASV GPC, Z, and/or NP (Josiah strain). Literature opined that those vaccines that expresses either GPC or NP alone furnishes the best protection level in cynomolgus macaques against a homologous virus challenge (i.e., challenge with the Josiah strain LASV) [135,137]. In cynomolgus macaques, MV-LASV was equally tested using varying doses, routes, and with heterologous challenge [138]. It was observed that the vaccine caused induction of lasting immune reactions against different LASV strains (lineages II and VII) and provokes T cell reactions against GPC and NP [139].

Moreso, tremendous progress has been made in adenovirus vector-based platforms development (for instance, ChAd3, ChAdOx1, ChAd63) as vaccine vectors, that can express antigens of several different pathogens including influenza and lASV, with few entering clinical trials in human [138,140]. The ChAdOx1 vaccine that expresses LASV GPC in Josiah strain has been revealed to be capable of producing an immune response and could cause induction of strong LASV-specific CD8 T cell (IFN and TNF-α, but low IL-2) and antibody reactions [141]. In addition, a single dosage of the ChAdOx1 LF vaccine has been reported to protect guinea pigs against lethal LASV (guinea pig-adapted Josiah strain) challenge [141].

Additionally, there was creation of RNA vaccine, a live attenuated ML29 vaccine by reassortment of the genomic RNA segments of Mopeia virus (MOPV) and LASV (Josiah strain [142]. For the creation of a single virion, there was reassortment of the L segment of the MOPV and the S segment of the LASV into a single particle. To perform this, there was coinfection of LASV and MOPV and thus isolation of putative reassortments by selecting only small plaques. The candidate vaccine ML29 was tested first using strain 13 inbred guinea pigs with a single subcutaneous (s.c.) dosage 30 days before challenge (103 PFU) and was revealed to furnish full protection against LASV challenge [143]. Carrion et al. [144] had reported that LASV challenge in marmosets orchestrated a fatal, systemic ailment. For the study of LF pathophysiology and vaccines’ effectiveness, Marmosets have been shown to be a great model as they share several histological features with humans. It was shown that ML29 single s.c. dose could bring about marmosets’ protection from LASV challenge and no animals showed any clinical symptoms [145].

Besides ML29, rLASV-GPC/CD (another live-attenuated LASV vaccine candidate) has equally been developed. The Codon deoptimization (CD) is a method which reduces expression of gene through swapping out the wild-type codons with less-preferred codons across the entire coding sequence of the target gene [146]. This technique has many merits including the fact that reverting to the wild-type sequence is very rare because CD is dependent on the introduction of many synonymous mutations in the gene sequence of the virus. Additionally, owing to the fact that CD do not culminate in amino acid alterations that alter the viral proteins, the virus containing the CD still maintain the same antigenic epitopes as the wild-type virus [147]. In addition, viruses can be swiftly produced by combination of de novo gene or genome synthesis with reverse genetics [148].

Even though ML29, rLASV-GPC/CD, and other live-attenuated vaccines show promising outcomes, a challenge with some LASV live-attenuated vaccine candidates is that a relatively high dosage is required to be efficacious. Significant negative effect can be observed using these high dosages of the LASV live-attenuated vaccines [147,149] as was seen in a similar platform for the development of EBOV candidate vaccines [150,151]. The usage of a vaccine that can cause the induction of undesirable effects in an area having poor care infrastructure could constitute a critical hurdle in the implementation of vaccine campaigns in West Africa [128].

## 5. Needs and Future Directions

### 5.1. Validation of Druggable Targets

Drug development beginning from screening stages to modulatory approval stage is costly and a time-demanding exercise that might take twelve to fifteen years and the cost has been evaluated to be more than $1 billion [152]. Notably, during clinical stages of development lowest success rates are observed. According to the report of David Szymkowski, Biotherapeutics Director with the Xencor Biopharmaceutical Company, the failure of compounds in the clinics might be ascribed to effectiveness or protection, and both of these are usually the direct outcomes of poor timely target validation [153].

Validation of target is critical and crucial in the process of drug discovery as drug development against a definite target is a huge commitment with respect to finance and time. Thus, experimental models are required to investigate if the selected target is contributory/involved in the concerned disease in humans. In vitro systems offer the prospect of testing and validating targets prior to the commitment of huge financial and transient resources to a promising lead compound. These systems allow the usage of reversible strategies that involves the regulation of gene expression in the concerned gene(s) and the monitoring of its action on the effectiveness of the candidate drug employing a myriad of biochemical and molecular endpoints. Consequently, this will furnish proof-of concept data which will precisely indicate if the promising target is involved in the drug action, hence reinforcing credence and lowering the possibility of costly failures of careless chosen candidates

### 5.2. Drug Repurposing

Drug repurposing also referred to as drug repositioning can be explained as a technique that involves the identification of pharmacological indications from old/earlier marketed/FDA licensed drugs and the use of redesigned drugs in the treatment of ailments apart from the drug’s original/intended therapeutic application. This entails the establishment of novel treatment for existing drugs, in addition to approved, discontinued, abandoned, and experimental drugs [154,155,156]. Unlike conventional drug discovery which is an exorbitant, burdensome, time-demanding, and risky process, drug repositioning has the advantage/capability of being used over conventional drug discovery as it mitigates the huge financial burden, prolonged time of manufacturing, and possibility of failure. It brings about a diminution in failure risk in which rate of failure of about 45 percent is linked with protection or toxicity challenges in convectional drug discovery programs including the merit of reducing the mean number of years in drug development time [157,158].

Following the evolution of cheminformatics/bioinformatics tools and accessibility of vast biological and structural repositories, repurposing of drugs has remarkably led to a reduction in the duration and financial burden associated with drug manufacturing with diminution in failure rates. Currently, the application of in silico methods as well as the evolution of structure-based drug design (SBDD) and artificial intelligence (AI) has further quickened the process of drug repurposing [159,160].

There are two approaches to drug repurposing viz: experiment-based method and in silico-dependent method. The experimental-dependent method also referred to as activity-based repurposing involves the screening of existing medications for a novel pharmacologic activity using investigations based on experiments. In this approach, target-based protein screening and cell/organism-based screen in ex vivo and/or in vivo disease models excluding any knowledge about the structure of proteins target are involved. Different methods of repositioning include target cell assay technique, screening technique, zoonotic model, and clinical approaches [161,162]. In silico repositioning on the other hand involves the searching of public databases having large libraries of drug employing computational and bioinformatics/cheminformatics devices. With this method, the achievement of promising biologically active molecules is dependent on the molecular interaction between the target protein and the drug molecule [163].

In silico technique has been accepted widely and recorded successes have been remarkable in drug discovery programs over the past years. In silico tools and techniques have been successfully used by several drug manufacturing companies and research laboratories in the discovery of a novel drug that differs in structure from various chemical spaces since substantial amounts of data on the structural chemistry of the biologically active compounds, pharmacophore models and protein’s structure are obtainable free online. Additionally, in silico repositioning has reduced time and financial burden associated with development as well as reduced risk of failure, unlike experimental-based approach [163,164].

Consequently, target validation and repurposing of existing drugs used in the management of LF such as ribavirin could be harnessed for the development of better therapeutics for curtailment of LF infection. At present, some cellular proteins likely to play a role in the replication and spread of LASV have been identified. For example, GSPT1 cellular protein currently identified might be a potential drug target, as CC-90009, a drug that specifically targets and degrades GSPT1, exhibited antiviral activity against LASV without cytotoxicity. Currently, CC-90009 is in phase 1 clinical development for the treatment of acute myeloid leukemia, raising the possibility of its repurposing as an antiviral against LASV [165]. Additionally, about 30 vaccines have been reported to be in the pre-clinical stage while 4 of them are currently undergoing clinical trials. The most promising candidates which started in 2019 were vesicular stomatitis virus-vectored vaccine and live-attenuated MV/LASV vaccine which had all progressed to clinical trials [166]. In addition, Wang et al. [158] have repurposed two hit compounds, lacidipine and phenothrin, that were identified as LASV entry inhibitors in the micromolar range. Mechanistically, these compounds were revealed to inhibit LASV entry by blocking low-pH-induced membrane fusion [158].

## 6. Challenges in the Management/Control of LF

The major clinical challenge in the management of LF is attributed to its high virulence and fatality rate that is further aggravated by the unspecific means of presentation (having similar signs like other fevers). Consequently, clinical investigation/diagnosis is mostly not plausible, particularly in our remote villages in the early presentation of this disease. The contagious nature of this disease creates a huge challenge for health workers and other caregivers mostly exposed to this ailment without protection before diagnoses and the establishment of barrier nursing. Its mode of contraction via fomite and aerosol poses another critical problem to all who have close contact with infected individuals [25]. It is also quite difficult to control the vectors. Since the dwelling place of these rodents is within and around homes and agricultural establishments, it becomes an onerous task to control. The control of these rodents using biological means such as the introduction of harmless predatory animals to rats such as cats within the endemic region optimistically has some prospects, although the aftermath later in life might be more unpleasant than the present. This will lead to disorganization of the ecosystem since there is no possibility that the cats will scrupulously eradicate the rodents alone. This line of thought may also be attributed to the use of chemicals (rat pesticides) which may not serve as an effective control measure. There is also the danger of the likelihood of the predators undergoing a genetic mutation and becoming transmuted into LASV carriers with the passage of time [25].

Another source of worry is the threat of complications that people who survived and those not having noticeable signs might suffer from. Robust evidence has indicated that subjects who survived and developed serious sensorineural hearing losses had distinctive poor speech differentiation and were not manageable using hearing aids [70]. However, the function of antioxidants and higher pressure of oxygen in mitigating loss in hearing and other neurologic problems are still speculative [167,168]. Moreso, there has been a report of imported cases of LF accidentally with severe consequences making the disease a global problem and not a clinical challenge restricted to the developing countries. The unavailability of safe vaccines and rapid diagnostic kits for diagnosis of the disease for nearly a decade after its identification has been a setback on its containment [25].

Lack of standard and inaccessibility of personal protective materials (PPM) particularly during the inception of the breakout of disease were critical challenges. This perhaps added immensely to the hospital-acquired spreading of the LASV to other medical personnel involved in the management of infected individuals. Thus, there is an urgent need to ensure that standard PPM is made available particularly masks, goggles and gloves to be put on during outbreak situations.

Furthermore, lack of proper awareness of the illness betwixt the people and medical personnel is another problem confronting the management of this disease culminating in fear of contracting the ailment. Consequently, this culminates in fear and tension among clinical personnel involved in the management of infected individuals. Difficulty in contact tracing is another challenge in combating this illness. There is a problem in tracing the origin of the disease to know the source of infection [24].

## 7. Discussion of Major Findings

LF is a severe viral hemorrhagic illness characterized by life-challenging, multiple organ failure. The lack of licensed medical countermeasures accentuates the need for the development of safe and efficacious treatment and prophylactics. Experiments using monoclonal antibodies and convalescent immunoglobulin formulations indicate that antibody-based treatment serves as an efficacious method of LF treatment [169]. The annual mortality rate of LF fever in West Africa has been estimated to be 5000. Political instability and war in hyperendemic regions have critically hindered its curtailment and management. Thus, vaccination is the most feasible control measure [170].

The curtailment of human contact with the zoonotic vector of LF is a crucial index in the prevention of LF, however, such extensive contact control seems impracticable currently in the West Africa endemic zones. Thus, the provision of vaccines for usage in communities and hospitals is of public health importance because vaccination is mostly the feasible control measure [170]. Mounting evidence had revealed that a vaccinia virus presenting the LASV glycoprotein had shown protection to four NHP against lethal challenge with LASV [171]. Following this study was another work that indicated that single injection of a vaccine presenting LASV glycoprotein full-length LASV protected against LF in primates, with or without NP expression [170]. The critical problems are how to surmount the political, scientific, and economic hurdles confronting the production of a vaccine for human usage. G-protein has been reported to confer protection with unknown duration but inclusion of NP had been suggested to give better protection in terms of duration even though but it is currently uncertain if the NP as a vaccine might probably promote the infection [172]. Proper allocation of financial resources in addition to the utilization of novel vaccine technologies offers hope that vaccines will soon be available for clinical evaluations, although, the problem of carrying out clinical investigations in West African endemic zones and political instability still poses a huge setback [33].

The LASV genome’s high rate of heterogenicity poses a serious problem in the manufacture of an efficacious pan-LASV vaccine and immunotherapeutic curtailment regimen. Ideally, any vaccination or antibody-dependent therapeutic should give defense against all strains of LASV. Previous studies indicated that four strains of LASV have been established, three is found in Nigeria which includes Lineages I, II, and III, while lineage IV is seen in many other West African countries including Sierra Leone, Guinea, and Liberia [84]. Additionally, many apparent lineages have been suggested as circulating strains in Mali and Côte d’Ivoire [173] (lineage V), Nigeria (lineage VI), and Togo [174] (lineage VII). Some human monoclonal antibodies exhibiting broad cross-neutralizing effect have been elucidated despite these genetic differences [175]. Hyperimmune sera from Virus-like particle-based immunized animals indicated efficacious counteracting effects opposed to homologous Josiah LASV strain (lineage IV) with the use of authentic LASV neutralization assays, and equally has the potential of nullifying the infectivity of a clinical isolate obtained from lineage II currently gotten from a LASV infected individual during LF outbreak in Nigeria in the 2018 [176]. Discovery by another pseudotype-dependent neutralization evaluation showed that it is promising that the immunization method employing glycoprotein isolated from the Josiah prototypic strain demonstrated widely reactive antibodies with the ability to halt the entry of the five distinct lineages of LASV [169].

Environmental factors have been reported to have serious impacts on the control of LF ailment, although the exact course of LASV transmission is yet to be decisively confirmed. For example, studies on environmental samples of LASV are scarce in order to point out the interface with the greatest challenge in transmissibility between rats and humans. The comprehension of the factors that promote the tenacity and spreading of LASV via the external environment would be better appreciated [177].

Although several works have been conducted on Lassa genetics and epidemiology, little attention has been paid to the impact of the environment and ecosystem on Lassa transmissibility. The spreading, maintenance, and continuation of LASV are linked to conditions of the environment that are related to the zoonotic vector [178]. The rats gain entrance into conducive areas which are filthy and messy including compounds overgrown by weeds, exposed solid-waste dumpsite, and disordered household items [179]. These rodents are particularly attracted by waste dumpsites because of the presence of leftover foodstuff and this thus facilitates their reproduction [179]. Overpopulation/congestion in households and bad environmental hygiene are also other factors linked with the transmission of LASV [180].

The high cost of accommodation, house rents, and student hostels in urban areas results in overcrowding owing to insufficient accommodation, particularly in less developed and developing countries. This makes individuals more prone and at greater threat of harboring the zoonotic host of LASV infection. Lack of personal and environmental sanitation and improper disposal of solid waste promotes the infection of LASV, with a huge risk for later spreading and preservation of LASV [177]. Furthermore, the identification of more environmental promoters of LF transmission such as humidity, temperature, including climatic factors such as seasonality should not be neglected. For example, dry season and rainy seasons dynamics concerning human habits and the rodents have been implicated in the spreading of LASV [181]. In Nigeria, the cases of LF have been reported to be at peak during the dry season. These are very important indexes for future Lassa outbreaks and could assist in the identification of risky human connections to rats in zones conducive for Lassa-targeted public health interventions. Putting the aforementioned factors promoting the spreading of LF into consideration, there is an urgent need for legislation and enforcement of guidelines by policymakers to combat these problems [177].

Taking into consideration the increasing incidence of LF in the endemic areas, it is expedient to contemplate the manufacture of a substitute and more efficacious countermeasures opposed to LASV infection via necessary and coordinated public-health methods that will earmark all Lassa possible routes of transmission and maintenance. Subsequent outbreaks and spreading of Lassa to regions previously with no record of Lassa can be tracked following scientific experiences and lessons garnered from the different breakout of Lassa and current progress in throughput genomics and mathematical modeling. Hamam and colleagues have documented different mathematical modelling for the control of L epidemic disease including the Stochastic Modelling [182]. Literature had shown that Okolo and colleagues developed a mathematical model for LF control by isolation and treatments [183]. They demonstrated that disease-free equilibrium is locally asymptotically. In addition, Marien and colleagues derived another mathematical model which is dependent on field data for the control of rats to combat LF and recommended continuous control or vaccination of the rats as only plausible policy [184]. Integrated approach as well as holistic and proactive strategies using One Health strategic approach is most reasonable, feasible, and effective methods [177]. This approach recognizes the interconnection of human health, animals, plants, and their common environment [185]. Thus, it provides superior and exhaustive data needed to handle the composite interaction of social, environmental, and ecological predictors of re-appearing infections of viral origin like LF [186]. This method will help investigators and legislators in tackling community health challenges created by Lassa explicitly and effectively via collaborative harmonization throughout relevant sectors and science disciplines. Thus, the comprehension of the biological, environmental, and social-economical predictor of LF upshot and transmission is vital to its control at community, state, and national levels [177].

## 8. Conclusions

Indisputably, this review has established that LF is a critical rodent-borne ailment that has witnessed epidemiological proportion and progression in the West Africa sub-region wherein soaring endemicity had been reported. The importance of this in public health cannot be overemphasized. The remarkable rise in the transborder traffic and international travels increases the likelihood of the transmission of the LASV to other zones within Africa and beyond the continent besides the probable periodic outbreaks of the disease within the region. The political instability and limited resources available for the health care delivery system characteristic of West African countries might continue to be a setback to the management of both emerging and currently devastating contagious ailments in the zone. Notwithstanding, the provision of properly furnished infectious disease laboratories and research centers including sufficient training of health care workers and other medical personnel would help in the quick investigation and treatment of highly infectious ailments such as LF and would thus help in preventing probable outbreaks. Additionally, in the endemic region, ribavirin should be provided in hospitals and health centers especially in the rural communities as this would be of assistance in the control of LF disease particularly at onset. This study is important because it x-rayed the pathogenesis and epidemiology of LF, its management and appraises the associated health challenges and the hurdles in combating the disease. Besides, effective preventive control measures, vaccine development, as well as repurposing of the existing drug like ribavirin using in silico-based approach have been suggested as plausible means of curtailing the dreaded disease.

## Figures and Tables

**Table 1:** Summary of previous studies/trials and details of possible treatments/therapies.

Drug	Type of Drug	Mechanism of Action	In Vitro Activity for Lassa	Animal Efficacy for Lassa	NHP Efficacy for Lassa	Investigated in Humans	Investigated in Humans Infected with Lassa	Precautions	Side Effects	Approval Status	Remarks	References
Ribavirin	Analogue of guanosine	Broad- spectrum of antiviral effect. Mode of action varies depending on the virus [116].	Yes	Yes. Administration of 160 mg/kg per day. Post-infection from days 4 to 11 markedly elongated survival in 20% of mice infected with LASV.	-	Yes. Investigated using different viruses like HCV, VHFs, etc.	Yes. Most potent Within 6 days Post-infection.	Pregnancy lactation (teratogenic and embryotoxic in rodents).	Dose-dependent hemolysis, seen in about 20% of infected individuals normally culminating in a modest diminution in hematocrit.	Yes, for VHFs.	Found to have a cell-protective activity in mice in comparison to Favipiravir. No evidence to corroborate its post-exposure prophylactic treatment for LF. Ribavirin has not shown a strong effect on advanced LF.	[17,60,98,116,117,118]
Favipiravir	Analogue of purine nucleoside, broad- spectrum effect against RNA viruses.	Inhibition of the viral (RdRp)	Yes	Yes. Post infection intake of 300 mg/kg daily from days 4 to 11 prevents death in 100% infected mice. Potent even when given 9 days post-infection in guinea pigs [101].	-	Stage-2 concluded (influenza) and stage-3 in progress (influenza).	No	Pregnancy lactation. Contraception is required at the end of treatment in women of childbearing age.	-	Yes, in Japan for novel and re-emerging influenza viruses.	Favipiravir and Ribavirin interact synergistically in vitro, prolonged survival rate and survival time when combining suboptimal doses in vivo.	[98,100,119,120,121]
CP	Blood products	Provokes passive immunization	Yes. It shows protection in monkeys studies.	Yes. Early intake provides protection in a guinea pig model.	Human CP protected recipient monkey.	Yes	Yes. Discordant reports. No activity limited activity however geographically matched plasma seems to be more efficacious.	The threat of transfusion transmitted infections, convalescent patients needed for donation.	Effect of transfusion reactions. Bloodborne viruses.	NA	Insufficient high-quality studies (i.e., randomized clinical trials) short window of efficacy in combination with ribavirin, has been revealed to be protective in NHP infected with LASV.	[17,86,110,113,117,122]
IFN alfacon-1	Non-naturally occurring bio-engineered alpha interferon.	Regulation of host innate immune responses.	No	No. Treatment remarkably protected PCV infected hamster model from death, extended the survival of those that died over time, lowered virus titers.	Yes. In monkeys infected with LASV. Timely and effective immune responses and modulation of viral replication were linked with recovery while fatal infection was marked by weak immune responses and unregulated viral replication.	Yes, for HCV.	No	? Pregnancy	Administration in bolus results in systemic toxicity, leucopenia thrombocy topaenia reduced Hb, psychiatric adverse events.	Yes, for treatment of chronic hepatitis C.	A combination of suboptimal doses of ribavirin (5–10 mg/kg/day) with IFN alfacon-1 (5–10 µg/kg/day) was shown to have both additive and synergistic activity when given within 24 h post infection with PCV (an arenavirus) in hamsters. IFN alfacon-1 treatment was more efficacious when given intranasally in comparison to delivery by i.p. injection.	[119,123,124,125,126]

Legend: RdRp = RNA-dependent RNA polymerase, CP = Convalescent Plasma, IFN = Interferon; LASV = Lassa virus, i.p = intraperitoneally, PCV = Pichinde virus, Hb = Hemoglobin, HCV = hepatitis C virus; VHFs = Viral hemorrhagic fevers [127].

## Data Availability

Not applicable.
